# Supplementary material for: Occurrence and Characteristics of Carbapenem-Resistant Klebsiella pneumoniae Strains Isolated from Hospitalized Patients in Poland—A Single Centre Study
Source: Pathogens. 2022 Jul 29;11(8):859. doi: 10.3390/pathogens11080859 (PMC9416609; doi:10.3390/pathogens11080859)
Supplement: Supplementary file 1 [file pathogens-11-00859-s001.zip › pathogens-1808450-tables.pdf]

Supplementary Tables S1 – S5

Table S1. The number of analyzed clinical materials in 2020, taking into account the hospital depatment and the type of material

Departments \ Materials	URINE	BLOOD	RT	WOUND	CSF	RS	STOOL	Σ
I	120	901	872	169	16	271	13	2362
II	112	12	0	527	26	4	6	687
III	54	63	1	347	0	0	7	472
IV	7	13	0	93	0	0	3	116
V	502	1694	178	52	0	279	60	2765
VI	342	204	17	19	0	0	0	582
VII	90	820	357	59	0	0	0	1326
VIII	48	61	36	71	15	31	0	262
IX	301	482	147	12	73	0	0	1015
X	271	251	75	39	0	0	0	636
XI	11	6	0	0	0	0	0	17
XII	304	467	2	81	41	1	0	896
XIII	125	297	68	34	3	0	20	547
XIV	120	175	99	535	0	226	9	1164
XV	375	198	0	95	0	8	5	681

Σ	2782	5644	1852	2133	174	820	123	13528

RS - rectal swabs for CPE carrier; RT – respiratory tract; CSF - cerebrospinal fluid; I- Department of Anaesthesiology and Intensive Care, II- Department of Pediatric Surgery, III- Department of General and Vascular Surgery, IV- Department of Plastic Surgery, V- Department of Internal Medicine, VI- Department of Endocrinology, Diabetology and Internal Medicine, VII- Department of Cardiology, VIII- Department of Neurosurgery, IX- Department of Neurology with the Stroke Division, X- Department of Rheumatology and Internal Medicine, XI- Department of Rehabilitation, XII- Department of Hospital Emergency, XIII- Department of Toxicology and Internal Medicine, XIV- Department of Orthopedic Surgery, XV- Department of Urology and Urological Oncology

Table S2. The number of *E. coli*, *K. pneumoniae* and *E. cloacae* isolates resistant to IMP, MEM or ETP

**Antibiotic**	*E. coli*	*K. pneumoniae*	*E. cloacae*
**Imipenem**	2	32	4
**Meropenem**	0	37	1
**Ertapenem**	21	58	36

Table S3. The number of CRE isolates, taking into account the hospital departments and the type of material**.**

	Urine	Blood	RT	Wound	RS
I	4	7	9	2	1
II	3	-	-	5	1
III	1	1	-	14	-
IV	1	-	-	-	-
V	8	1	1	-	5
VI	1	2	-	-	-

VII	-	-	-	1	-
VIII	-	-	2	1	-
IX	3	-	2	-	-
X	-	-	-	1	-
XII	5	-	-	1	1
XIII	5	-	-	1	-
XIV	6	-	-	4	8
XV	4	-	-	1	-

RS - rectal swabs for CPE carrier; RT – respiratory tract; I- Department of Anaesthesiology and Intensive Care, II- Department of Pediatric Surgery, III- Department of General and Vascular Surgery, IV- Department of Plastic Surgery, V- Department of Internal Medicine, VI- Department of Endocrinology, Diabetology and Internal Medicine, VII- Department of Cardiology, VIII- Department of Neurosurgery, IX- Department of Neurology with the Stroke Division, X- Department of Rheumatology and Internal Medicine, XI- Department of Rehabilitation, XII- Department of Hospital Emergency, XIII- Department of Toxicology and Internal Medicine, XIV- Department of Orthopedic Surgery, XV- Department of Urology and Urological Oncology

Table S4. The number of *K. pneumoniae* isolates including the MIC values for imipenem and meropenem

**MIC values**												
MIC for IMP and MEM (mg/L)	≤0.25	0.5	0.75	1	1.5	2	3	4	8	16	32	>32
The number of *K. pneumoniae* isolates for imipenem	315	45	2	17	2	3	3	5	10	4	8	2
The number of *K. pneumoniae* isolates for meropenem	356	21	0	1	1	0	0	9	10	12	2	4

Table S5. The results of phenotypic, immunochromatographic and Carba NP tests used to detect carbapenemases in studied *K. pneumoniae* isolates in 2020.

No.	Date	Material	Department	Identification	KPC	MBL	OXA-48	Cassette Test	CarbaNP
1	2020-06-22	RS	XIV	K. pneum	-	+	-	NDM	+
2	2020-06-22	RS	XIV	K. pneum	-	+	-	NDM	+
3	2020-08-28	RS	XIV	K. pneum	-	-	-	-	-
4	2020-08-28	RS	XIV	K. pneum	-	+	-	NDM	+
5	2020-06-17	RS	V	K. pneum	-	+	-	NDM	+
6	2020-09-29	RS	XII	K. pneum	-	+	-	VIM	+
7	2020-06-22	RS	V	K. pneum	-	+	-	NDM	+
8	2020-06-22	RS	V	K. pneum	-	+	-	NDM	+
9	2020-06-21	RS	I	K. pneum	-	+	-	NDM	+
10	2020-09-29	RS	II	K. pneum	-	+	-	VIM	+
11	2020-07-06	RS	V	K. pneum	-	+	-	NDM	+
12	2020-07-20	RS	V	K. pneum	-	-	-	-	-
13	2020-08-15	RS	XIV	K. pneum	-	+	-	NDM	+
14	2020-09-04	RS	XIV	K. pneum	-	-	-	NDM	+
15	2020-09-25	RS	XIV	K. pneum	-	-	-	NDM	+

16	2020-09-04	RS	XIV	K. pneum	-	-	-	-	-
17	2020-11-26	RT	IX	K. pneum	-	-	-	-	-
18	2020-01-23	RT	V	K. pneum	-	-	-	-	-
19	2020-12-26	RT	I	K. pneum	-	-	-	-	-
20	2020-11-09	RT	I	K. pneum	-	-	-	-	-
21	2020-12-25	RT	I	K. pneum	-	-	-	-	-
22	2020-06-17	Blood	I	K. pneum	-	+	-	NDM	+
23	2020-02-22	Blood	I	K. pneum	-	+	+	OXA-48, NDM	+
24	2020-07-04	Blood	I	K. pneum	-	+	-	NDM	+
25	2020-11-10	Blood	V	K. pneum	-	-	-	NDM	+
26	2020-12-25	Blood	I	K. pneum	-	-	-	-	-
27	2020-05-07	Urine	V	K. pneum	-	-	-	-	-
28	2020-04-02	Urine	XII	K. pneum	-	-	-	-	-
29	2020-07-07	Urine	XII	K. pneum	-	-	-	-	-
30	2020-01-13	Urine	XII	K. pneum	-	-	-	-	-
31	2020-06-22	Urine	IV	K. pneum	-	-	-	-	-
32	2020-01-23	Urine	IX	K. pneum	-	-	-	-	-
33	2020-08-07	Urine	XIV	K. pneum	-	-	-	-	-

34	2020-10-15	Urine	V	K. pneum	-	-	-	-	-
35	2020-10-15	Urine	VII	K. pneum	-	-	-	-	-
36	2020-06-15	Urine	XIV	K. pneum	-	+	-	NDM	+
37	2020-06-24	Urine	XIV	K. pneum	-	-	-	NDM	+
38	2020-09-08	Urine	XIV	K. pneum	-	-	-	NDM	+
39	2020-01-07	Urine	XIII	K. pneum	-	-	-	-	-
40	2020-09-24	Urine	V	K. pneum	-	+	-	NDM	+
41	2020-06-10	Urine	V	K. pneum	-	+	-	NDM	+
42	2020-05-25	Urine	XV	K. pneum	-	-	-	-	-
43	2020-05-11	Urine	III	K. pneum	-	+	-	VIM	+
44	2020-09-25	Urine	II	K. pneum	-	-	-	VIM	+
45	2020-12-10	Urine	XV	K. pneum	-	-	-	-	-
46	2020-12-01	Urine	XV	K. pneum	-	-	-	-	-
47	2020-11-24	Urine	XIII	K. pneum	-	-	-	-	-
48	2020-11-30	Urine	XIII	K. pneum	-	+	-	NDM	+
49	2020-11-22	Urine	IX	K. pneum	-	-	-	-	-
50	2020-11-30	Urine	IX	K. pneum	-	-	-	-	-
51	2020-08-08	Urine	VI	K. pneum	-	-	-	-	-

52	2020-05-29	Wound	III	K. pneum	-	-	-	-	-
53	2020-12-11	Wound	III	K. pneum	-	+	-	VIM	+
54	2020-11-15	Wound	XII	K. pneum	-	-	-	-	-
55	2020-01-15	Wound	X	K. pneum	-	-	-	-	-
56	2020-11-28	Wound	III	K. pneum	-	-	-	-	-
57	2020-11-28	Wound	III	K. pneum	-	+	-	NDM	+
58	2020-02-01	Wound	VIII	K. pneum	-	+	-	NDM	+

RS* - rectal swabs for CPE carrier; RT – respiratory tract; I- Department of Anaesthesiology and Intensive Care, II- Department of Pediatric Surgery, III- Department of General and Vascular Surgery, IV- Department of Plastic Surgery, V- Department of Internal Medicine, VI- Department of Endocrinology, Diabetology and Internal Medicine, VII- Department of Cardiology, VIII- Department of Neurosurgery, IX- Department of Neurology with the Stroke Division, X- Department of Rheumatology and Internal Medicine, XI- Department of Rehabilitation, XII- Department of Hospital Emergency, XIII- Department of Toxicology and Internal Medicine, XIV- Department of Orthopedic Surgery, XV- Department of Urology and Urological Oncology
