# Supplementary material for: Occurrence and Characteristics of Carbapenem-Resistant Klebsiella pneumoniae Strains Isolated from Hospitalized Patients in Poland—A Single Centre Study
Source: Pathogens. 2022 Jul 29;11(8):859. doi: 10.3390/pathogens11080859 (PMC9416609; doi:10.3390/pathogens11080859)
Supplement: Supplementary file 1 [file pathogens-11-00859-s001.zip › Supplementary Figure S1-S3.pdf]

Supplementary Figures S1-S3.

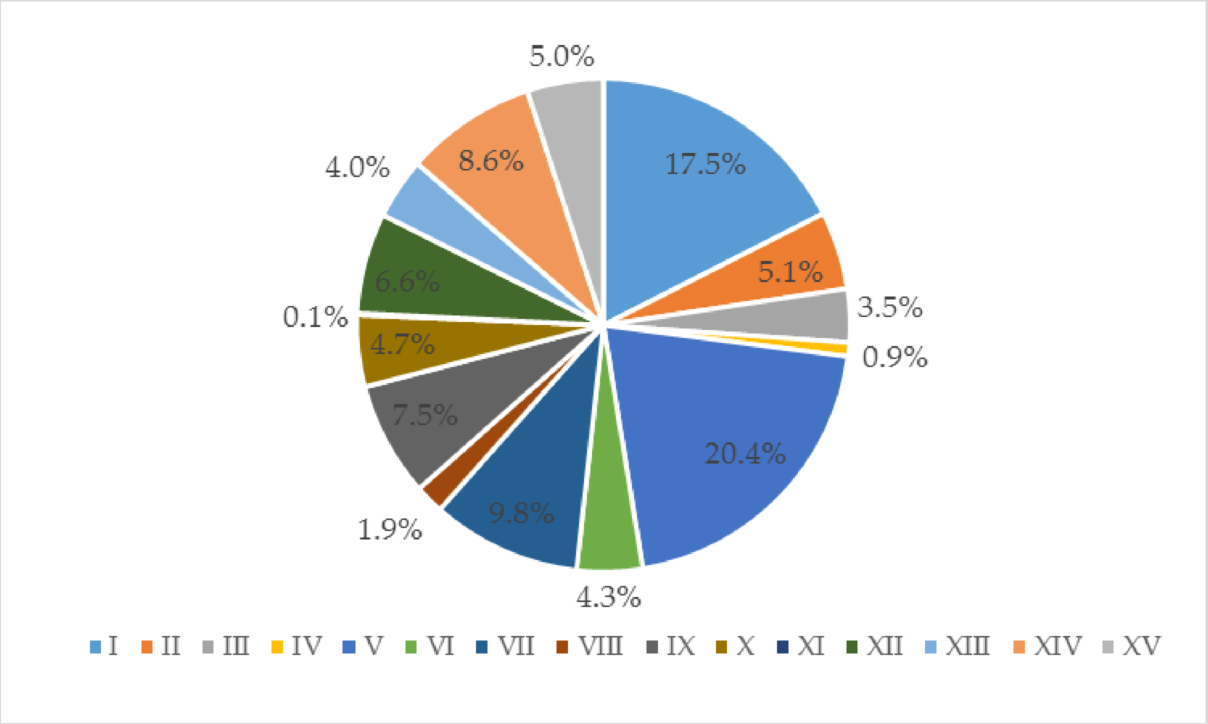

I- Department of Anaesthesiology and Intensive Care, II- Department of Pediatric Surgery, III- Department of General and Vascular Surgery, IV- Department of Plastic Surgery, V- Department of Internal Medicine, VI- Department of Endocrinology, Diabetology and Internal Medicine, VII- Department of Cardiology, VIII- Department of Neurosurgery, IX- Department of Neurology with the Stroke Division, X- Department of Rheumatology and Internal Medicine, XI- Department of Rehabilitation, XII- Department of Hospital Emergency, XIII- Department of Toxicology and Internal Medicine, XIV- Department of Orthopedic Surgery, XV- Department of Urology and Urological Oncology

Figure S1. Percentage of the analyzed clinical materials, taking into account the hospital department.

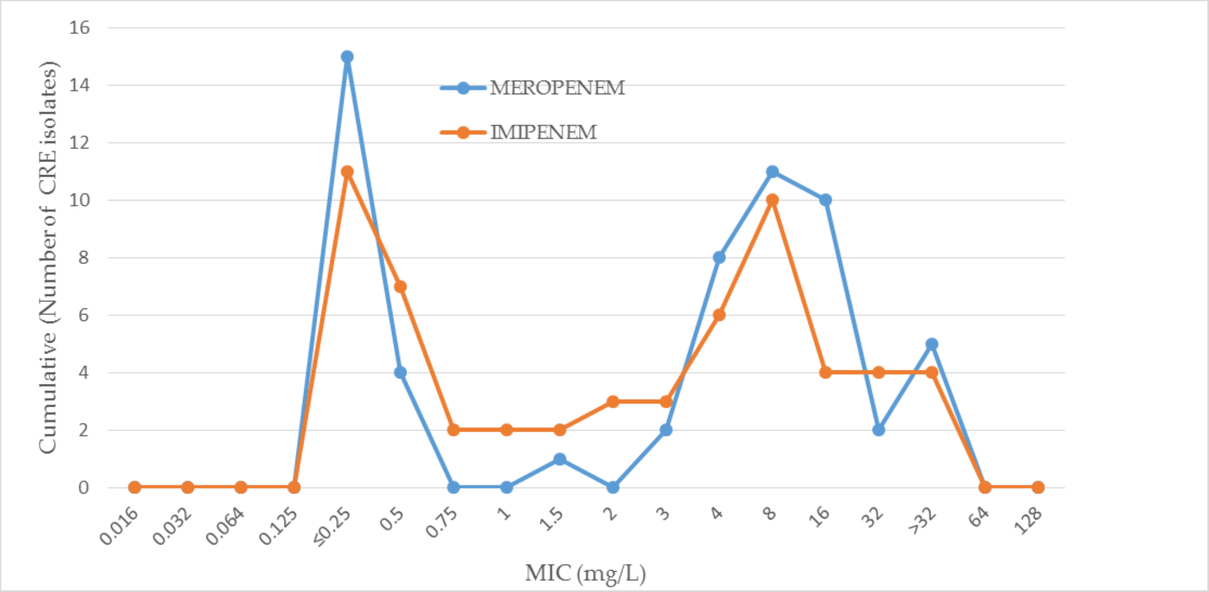

Figure S2. Cumulative MIC of K. pneumoniae CRE against carbapenems.

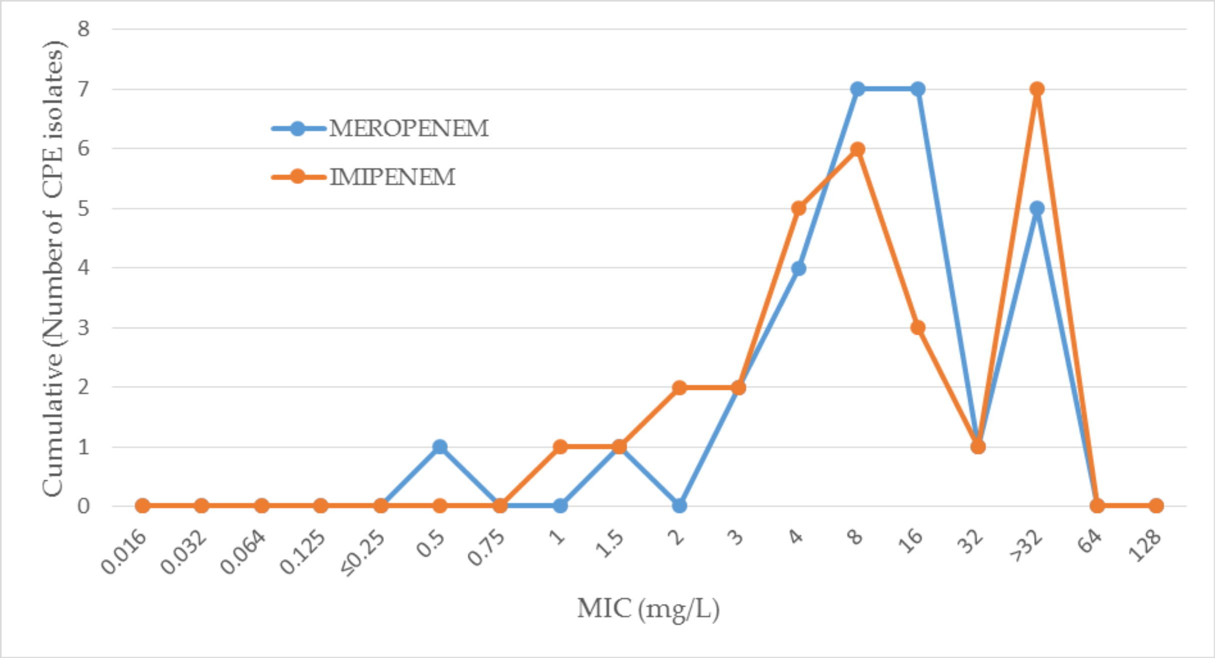

Figure S3. Cumulative MIC of K. pneumoniae CPE against carbapenems.
